# An integrative analysis of an lncRNA–mRNA competing endogenous RNA network to identify functional lncRNAs in uterine leiomyomas with RNA sequencing

**DOI:** 10.3389/fgene.2022.1053845

**Published:** 2023-01-04

**Authors:** Fanfei Meng, Yijing Ji, Xinyu Chen, Yuan Wang, Maofang Hua

**Affiliations:** Department of Gynecology, Lianyungang Maternal and Child Health Hospital, Lianyungang, China

**Keywords:** uterine leiomyomas, long non-coding RNA, mRNAs, high-throughput sequencing, miRNAs

## Abstract

**Objective:** To explore the functions of mRNAs and lncRNAs in the occurrence of uterine leiomyomas (ULs) and further clarify the pathogenesis of UL by detecting the differential expression of mRNAs and lncRNAs in 10 cases of UL tissues and surrounding normal myometrial tissues by high-throughput RNA sequencing.

**Methods:** The tissue samples of 10 patients who underwent hysterectomy for UL in Lianyungang Maternal and Child Health Hospital from January 2016 to December 2021 were collected. The differentially expressed mRNAs (DEmRNAs) and lncRNAs (DElncRNAs) were identified and further analyzed by Gene Ontology (GO) and Kyoto Encyclopedia of Genes and Genomes (KEGG) pathway enrichment analysis. The protein–protein interaction network (PPI) was constructed in Cytoscape software. Functional annotation of the nearby target cis‐DEmRNAs of DElncRNAs was performed with the Database for Annotation, Visualization, and Integrated Discovery (DAVID) (https://david.ncifcrf.gov/). Meanwhile, the co-expression network of DElncRNA–DEmRNA was constructed in Cytoscape software.

**Results:** A total of 553 DElncRNAs (283 upregulated DElncRNAs and 270 downregulated DElncRNAs) and 3,293 DEmRNAs (1,632 upregulated DEmRNAs and 1,661 downregulated DEmRNAs) were obtained. GO pathway enrichment analysis revealed that several important pathways were significantly enriched in UL such as blood vessel development, regulation of ion transport, and external encapsulating structure organization. In addition, cytokine–cytokine receptor interaction, neuroactive ligand–receptor interaction, and complement and coagulation cascades were significantly enriched in KEGG pathway enrichment analysis. A total of 409 DElncRNAs–nearby-targeted DEmRNA pairs were detected, which included 118 DElncRNAs and 136 DEmRNAs. Finally, we found that the top two DElncRNAs with the most nearby DEmRNAs were BISPR and AC012531.1.

**Conclusion:** These results suggested that 3,293 DEmRNAs and 553 DElncRNAs were differentially expressed in UL tissue and normal myometrium tissue, which might be candidate-identified therapeutic and prognostic targets for UL and be considered as offering several possible mechanisms and pathogenesis of UL in the future.

## Introduction

Uterine leiomyoma (UL), as one of the most common benign tumors in women, arises from the non-neoplastic proliferation of smooth muscle cells within the myometrium (Segars and Al-Hendy, 2017; Yuan et al., 2021). UL occurs in more than 70% of women before menopause, with an incidence of up to 20%–25% in women of reproductive age (Lv et al., 2008; Giuliani et al., 2020). Accumulating clinical evidence has demonstrated that UL has caused a lot of health problems, including uterine bleeding, dysmenorrhoea, repetitive pregnancy loss, menorrhagia, infertility, pelvic pressure, and pain (El et al., 2021; Mathew et al., 2021). Therefore, it is urgent to find new etiological and pathological features of UL to improve the diagnostic efficiency of UL (Yuan et al., 2021). To date, little is known about the pathogenesis of UL, leading to the lack of a valid medical treatment (Falahati et al., 2021). However, there is considerable evidence that estrogen plays a major role in the proliferation of these benign tumors (Ulin et al., 2020). While a strong correlation between ovarian hormone levels or age and the development of UL was found, recent studies of UL have identified novel genes or pathways suggesting the differential expression of mRNAs and lncRNA of this disease progression in order to improve the early detection of tumor and early intervention and treatment of UL (Nothnick, 2016; Falahati et al., 2021).

With the development of high-throughput sequencing, novel bioinformatics approaches, and corresponding experimental validation, a broad spectrum of mRNAs and lncRNAs have been suggested to play an important role in various biological processes (Klapproth et al., 2021; Lin et al., 2022). Long non-coding RNAs (lncRNAs) are a type of long non-coding RNA molecules, more than 200 nucleotides in length, which limited their protein-encoding potential (Zhao et al., 2021). Functionally, accumulating evidences have implicated that lncRNAs are involved in various physiological processes, such as human genome imprinting, chromatin remodeling, X chromosome silencing, epigenetic regulation, and cell cycle and differentiation, and that the abnormal expression and dysfunction of lncRNAs play an functional role in the occurrence and development of a variety of diseases (Wu et al., 2021; Jain et al., 2022). Mechanistically, more and more studies have shown that lncRNAs could participate in gene expression by recruiting the chromosome remodeling complex into a gene promoter (Liu et al., 2021; Ping et al., 2021; Najafi et al., 2022). Also, several lncRNAs fulfill their roles by serving as sponges to arrest miRNA function (Ping et al., 2021). In addition, they also directly interact with some important proteins to augment or attenuate their function (Liu et al., 2021). In addition, the abnormal expression or functional changes in lncRNA expression have become important biological regulators for understanding the molecular mechanisms of these diseases and identifying effective diagnostic biomarkers and therapeutic targets (Hu et al., 2021; Zhao et al., 2021). However, little information available in literature studies about the association between lncRNAs and the progression of UL has yet been elucidated.

In the present study, it is aimed to explore the functions of mRNAs and lncRNAs in the occurrence of uterine leiomyomas (ULs) and further clarify the pathogenesis of UL by detecting the differentially expressed lncRNAs (DElncRNAs) and mRNAs (DEmRNAs) in 10 cases of UL tissues and surrounding normal myometrial tissues by high-throughput RNA sequencing. The results found that a total of 553 DElncRNAs (283 upregulated DElncRNAs and 270 downregulated DElncRNAs) and 3,293 DEmRNAs (1,632 upregulated DEmRNAs and 1,661 downregulated DEmRNAs) were obtained. This study could represent a novel proposal to increase the understanding of the pathogenesis and molecular targeted therapy of UL.

## Materials and methods

### Ethical compliance

The experiment complied with the Ethics Committee of Lianyungang Maternal and Child Health Hospital (LYG-ME202004), and written informed consent was obtained from each participant before performing surgical procedures.

### Patients and samples

A total of 10 UL pairs with tumor samples and surrounding normal myometrial tissue samples were obtained and chosen for high-throughput RNA sequencing at random from UL patients who underwent hysterectomy in Lianyungang Maternal and Child Health Hospital from January 2016 to December 2021. All of these patients with UL were free of treatment before surgery. Pathological diagnostics for UL were independently diagnosed by three experienced pathologists. Fresh tissue samples were immediately frozen in liquid nitrogen after resection from UL patients to protect the protein or RNA from degradation.

### RNA extraction and RNA sequencing

According to the manufacturer’s protocol, the total RNA was isolated and extracted from frozen UL tissues and surrounding normal myometrial tissues using the TRIzol reagent (Invitrogen, Carlsbad, CA, United States). One microgram of total RNA was reverse transcribed to high-quality cDNA using a PrimeScript RT Master Mix (Vazyme Biotech, Nanjing, China). With the Agilent 2100 Bioanalyzer, the concentration, integrity, and RNA integrity number (RIN) values of RNA were assessed. RNA sequencing was performed based on the Illumina HiSeq platform (Illumina, Inc., San Diego, CA, United States) in 151-bp sequencing mode. The RNA sequencing was performed with paired ends and 10 G depth. With Base Calling version 0.11.4 (http://www.bioinformatics.babraham.ac.uk/projects/fastqc/), the FASTQ sequence data were acquired from the RNA sequencing data. The Read QC tool in FastQC version 0.11.4 (http://www.bioinformatics.babraham.ac.uk/projects/ fastqc/) was used for the quality control of FASTQ data with Q > 30. Trimming of raw data was performed with cutadapt version 1.16 (http://cutadapt.readthedocs.io). Reads with low quality were removed to obtain the clean reads.

### Identification of DEmRNAs and DElncRNAs

In order to align the clean reads with the human reference genome, Ensembl GRCh38.p7 and HISAT2 version 2.1.0 were applied. The expression levels of mRNAs and lncRNAs were normalized and outputted with StringTie version 1.3.3b (http://ccb.jhu.edu/software/stringtie/). Fragments per kilobase of exon per million fragments mapped (FPKM) of lncRNAs and mRNAs were calculated with StringTie. With edgeR version 3.24 (http://www.bioconductor.org/packages/release/bioc/html/edgeR.html), both DEmRNAs and DElncRNAs were obtained with |log2FC| > 1 and a *p* value < 0.05. By using the R package “pheatmap,” hierarchical clustering analysis of DElncRNAs and DEmRNAs was conducted.

### Functional annotation of DEmRNAs

Gene Ontology (GO) and Kyoto Encyclopedia of Genes and Genomes (KEGG) pathway enrichment analyses were performed with the Database for Annotation, Visualization, and Integrated Discovery (DAVID) (https://david.ncifcrf.gov/). Statistical significance was assumed when *p* < 0.05.

### Construction of protein–protein interaction networks

The top 100 upregulated DEmRNAs and 100 downregulated DEmRNAs were submitted to the STRING (https://www.string-db.org/) database. Then, PPI networks were visualized by Cytoscape software (version 3.5.0, http://www.cytoscape. org).

### Cis-nearby-targeted DEmRNAs of the DElncRNAs

DEmRNAs transcribed within a 100-kb window upstream or downstream of DElncRNAs were searched, which were defined as cis-nearby-targeted DEmRNAs of DElncRNAs, to obtain the targeted DEmRNAs of DElncRNAs with cis-regulatory effects. The networks were visualized by Cytoscape software. Functional annotation of the cis-nearby-targeted DEmRNAs of the DElncRNAs was conducted with DAVID.

### DElncRNA–DEmRNA co-expression networks

To further examine the potential roles of DElncRNAs and DEmRNAs in UL, the DElncRNA–DEmRNA co-expression networks were constructed. DElncRNA–DEmRNA pairs with an absolute value of PCC > 0.95 and *p* < 0.01 were defined as co-expressed DElncRNA–DEmRNA pairs. By using Cytoscape, the co-expressed DElncRNA–DEmRNA networks were visualized. Functional annotation of the DEmRNAs co-expressed with DElncRNAs was performed with DAVID. A value of *p* < 0.05 was set as the cut-off for significance.

## Results

### DElncRNA and DEmRNA expression profiling in uterine leiomyomas

**FIGURE 1:**
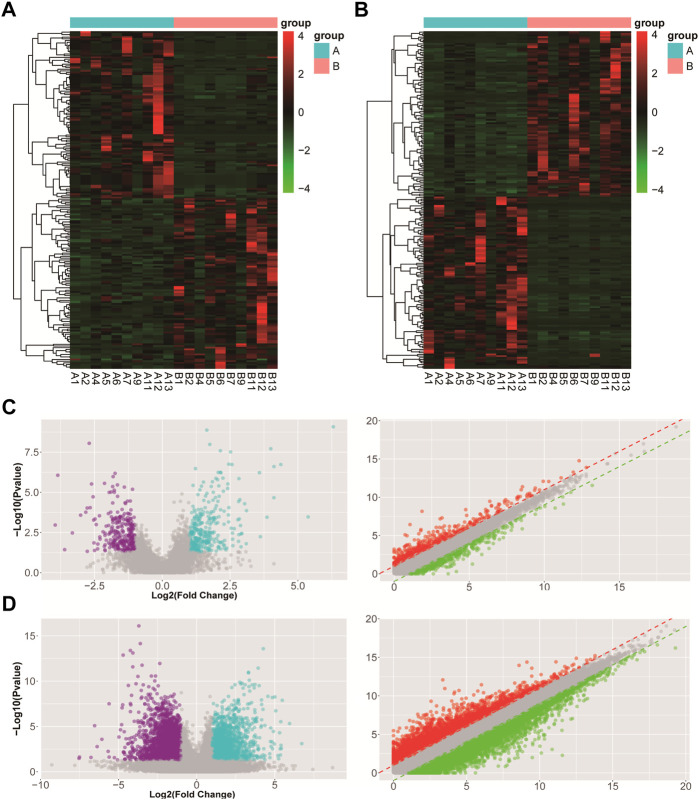
DElncRNA and DEmRNA expression patterns in uterine leiomyoma tissues are related to those in surrounding normal myometrial tissues. **(A)** Hierarchical cluster analysis of all differentially expressed DElncRNAs. **(B)** Hierarchical cluster analysis of all differentially expressed DEmRNAs; each column represents a sample, and each row represents a lncRNA and mRNA. The color scale indicates relative expression: upregulation (red) and downregulation (blue). A represents the uterine leiomyoma tissue group, and B represents the surrounding normal myometrial tissue group. **(C)** Volcano and scatter plots demonstrate differential expression of DElncRNAs between two different conditions. **(D)** Volcano and scatter plots demonstrate differential expression of DEmRNAs between two different conditions; red points indicate upregulated expression, while green points indicate downregulated expression. The values plotted on the *X* and *Y* axes are the averaged normalized signal values of each group (log2 scaled). FC ≥ 2 and *p* ≤ 0.05 were regarded as the differentially expressed DElncRNAs and DEmRNAs.

A total of 553 DElncRNAs (283 upregulated DElncRNAs and 270 downregulated DElncRNAs) and 3,293 DEmRNAs (1,632 upregulated and 1,661 downregulated DEmRNAs) were found in UL tissue samples. Furthermore, the top 10 upregulated and downregulated DElncRNAs and DEmRNAs are shown in Table 1 and Table 2, respectively. Hierarchical clustering analysis of the top 100 upregulated and downregulated DElncRNAs and DEmRNAs between UL samples and surrounding normal myometrial tissue samples is presented in Figure 1A and Figure 1B, respectively. The volcano and scatter plots displayed the variation in DElncRNA and DEmRNA expression between UL groups and surrounding normal myometrial tissue groups (Figure 1C and Figure 1D).

**TABLE 1:** Top 10 upregulated and downregulated DEmRNAs in uterine leiomyomas.

Gene_ID	Symbol	log2 fold change	*p* value	Regulation
ENSG00000142549	IGLON5	4.32	1.64E-15	Up
ENSG00000228203	RNF144A-AS1	2.13	1.23E-13	Up
ENSG00000153404	PLEKHG4B	4.23	7.86E-13	Up
ENSG00000183778	B3GALT5	3.38	2.14E-12	Up
ENSG00000260296	AC095057.3	1.85	8.65E-11	Up
ENSG00000235996	AL136090.1	3.52	1.09E-10	Up
ENSG00000140557	ST8SIA2	3.62	1.11E-10	Up
ENSG00000033122	LRRC7	2.78	2.71E-10	Up
ENSG00000275139	AL133492.1	2.65	4.14E-10	Up
ENSG00000157388	CACNA1D	2.20	5.00E-10	Up
ENSG00000127954	STEAP4	−3.57	5.72E-17	Down
ENSG00000137767	SQOR	−2.23	4.60E-13	Down
ENSG00000158104	HPD	−3.83	1.26E-12	Down
ENSG00000180914	OXTR	−4.64	1.73E-11	Down
ENSG00000154198	CYP4Z2P	−3.50	2.34E-11	Down
ENSG00000239265	CLRN1-AS1	−2.96	3.67E-11	Down
ENSG00000237510	GPAT2P1	−3.19	4.26E-11	Down
ENSG00000186160	CYP4Z1	−2.86	6.76E-11	Down
ENSG00000137699	TRIM29	−2.53	1.03E-10	Down
ENSG00000162591	MEGF6	−2.73	1.27E-10	Down

Count: the number of DEGs that hit in the term.

**TABLE 2:** Top 10 upregulated and downregulated DElncRNAs in uterine leiomyomas.

Gene_ID	Symbol	log2 fold change	*p* value	Regulation
ENSG00000272002	AC010904.2	1.64	1.35E-09	Up
ENSG00000272371	AL591167.1	1.75	1.04E-08	Up
ENSG00000233723	LINC01122	4.00	1.95E-08	Up
ENSG00000259828	AL355596.1	4.37	1.87E-07	Up
ENSG00000226917	LINC01276	2.57	1.87E-07	Up
ENSG00000270147	AC068620.3	1.23	3.33E-07	Up
ENSG00000251536	AC055717.1	2.14	5.72E-07	Up
ENSG00000255628	AC140847.1	2.06	5.79E-07	Up
ENSG00000262772	LINC01977	3.58	6.03E-07	Up
ENSG00000243903	AC138057.1	2.16	6.05E-07	Up
ENSG00000246430	LINC00968	−2.69	8.95E-09	Down
ENSG00000230746	AC006007.1	−3.85	8.74E-07	Down
ENSG00000250742	LINC02381	−1.81	1.06E-06	Down
ENSG00000233117	LINC00702	−2.13	2.78E-06	Down
ENSG00000283897	AC011416.4	−2.61	3.04E-06	Down
ENSG00000272235	AL590438.1	−1.65	3.46E-06	Down
ENSG00000261462	AC004023.1	−1.83	4.39E-06	Down
ENSG00000277152	AC110048.2	−1.10	6.50E-06	Down
ENSG00000233521	LINC01638	−1.77	7.40E-06	Down
ENSG00000238033	AC002480.4	−1.89	9.57E-06	Down

Count: the number of DEGs that hit in the term.

### Functional annotation analysis

**FIGURE 2:**
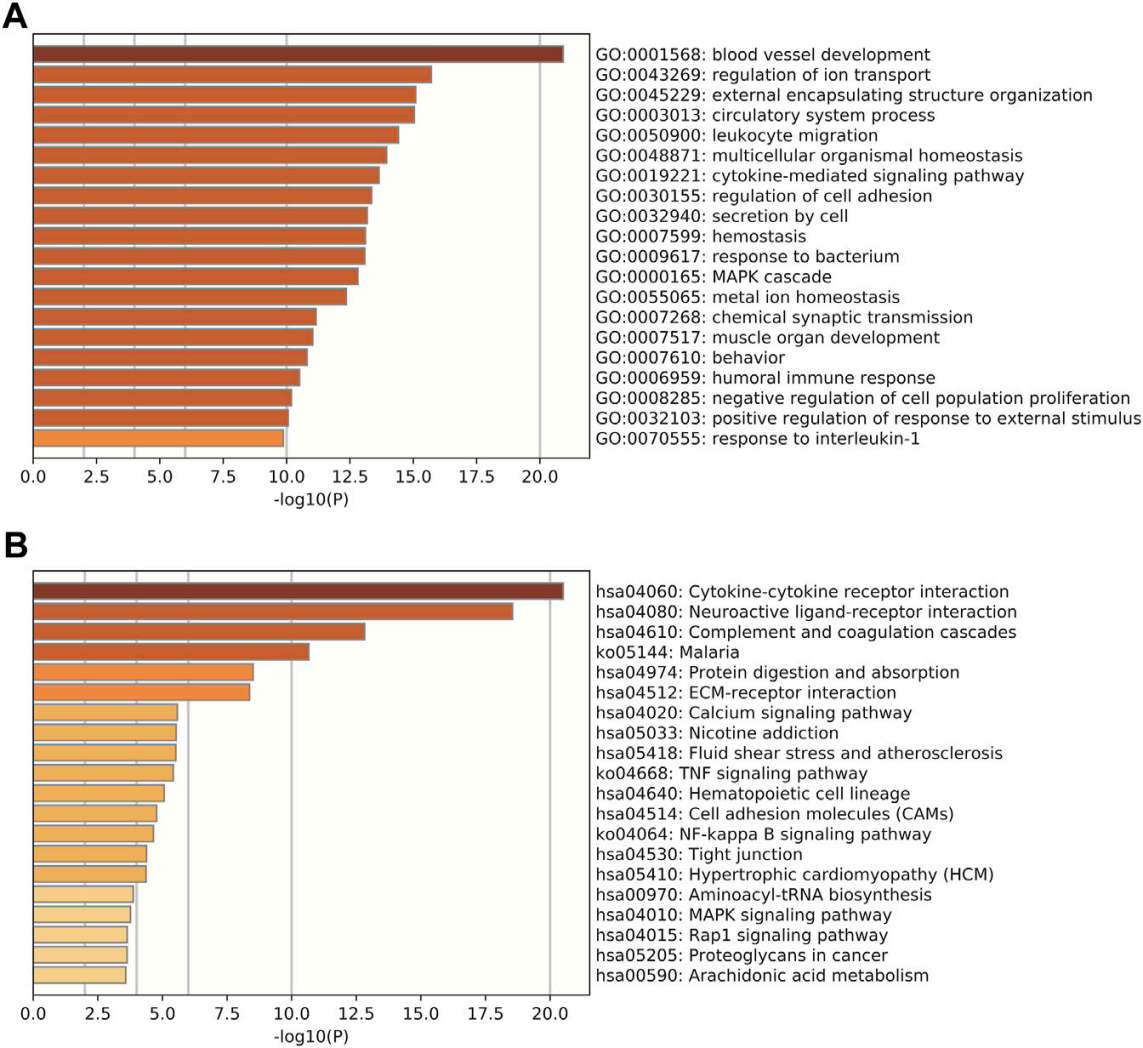
Functional annotation analysis for the validated DEmRNA gathering genes. **(A)** GO analysis. **(B)** KEGG pathway analysis. The vertical axis shows the annotated functions of the target genes. The horizontal axes show the enrichment score (−log10 transformed *p* value) and the gene number of each cluster, respectively. Only the top 10 significantly enriched clusters are included.

GO and KEGG pathway enrichment analyses were performed for the DEmRNAs. GO analysis revealed that mRNAs were downregulated in UL and mainly involved in a number of signaling pathways, including blood vessel development (*p* = 1.21 E-21), regulation of ion transport (*p* = 1.94E-16), and external encapsulating structure organization (*p* = 8.05E-16) (Figure 2A). KEGG pathway analysis revealed that the deregulated mRNAs were primarily enriched in signaling pathways associated with cytokine–cytokine receptor interaction (*p* = 3.10E-21), neuroactive ligand–receptor interaction (*p* = 2.67E-21), and complement and coagulation cascades (CAMs) (*p* = 1.50E-18) (Figure 2B).

### Protein–protein interaction networks

**FIGURE 3:**
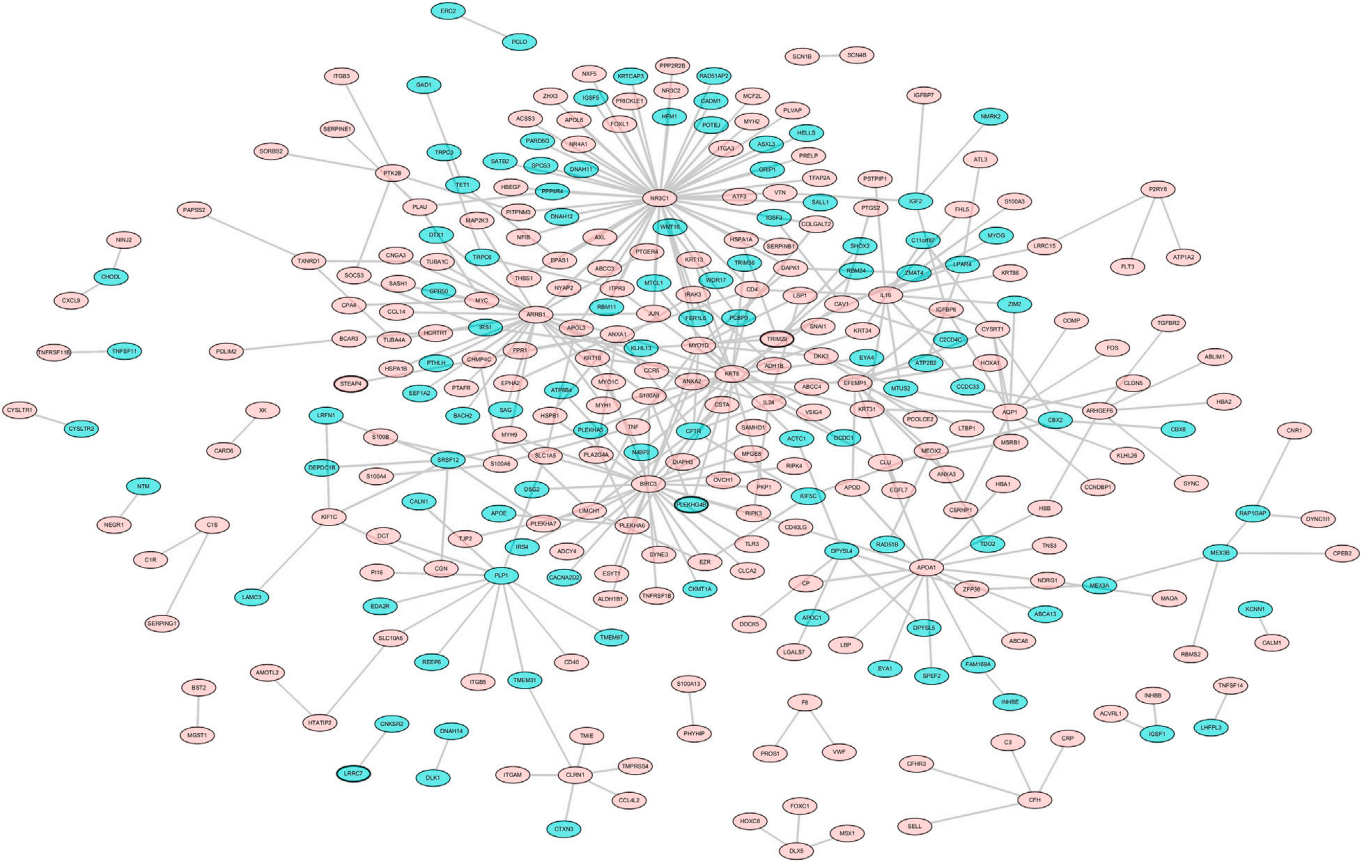
Protein–protein interaction (PPI) network analysis. The network comprising the top upregulated (red triangles) and downregulated lncRNAs (green inverted arrows) and their target mRNAs is presented.

PPI networks were constructed and included 313 nodes and 347 edges. NR3C1 (degree = 57), BIRC3 (degree = 27), ARRB1 (degree = 24), KRT8 (degree = 20), and APOA1 (degree = 15) were the top five hub proteins in the PPI networks (Figure 3).

### Cis-nearby-targeted DEmRNAs of DElncRNAs

**FIGURE 4:**
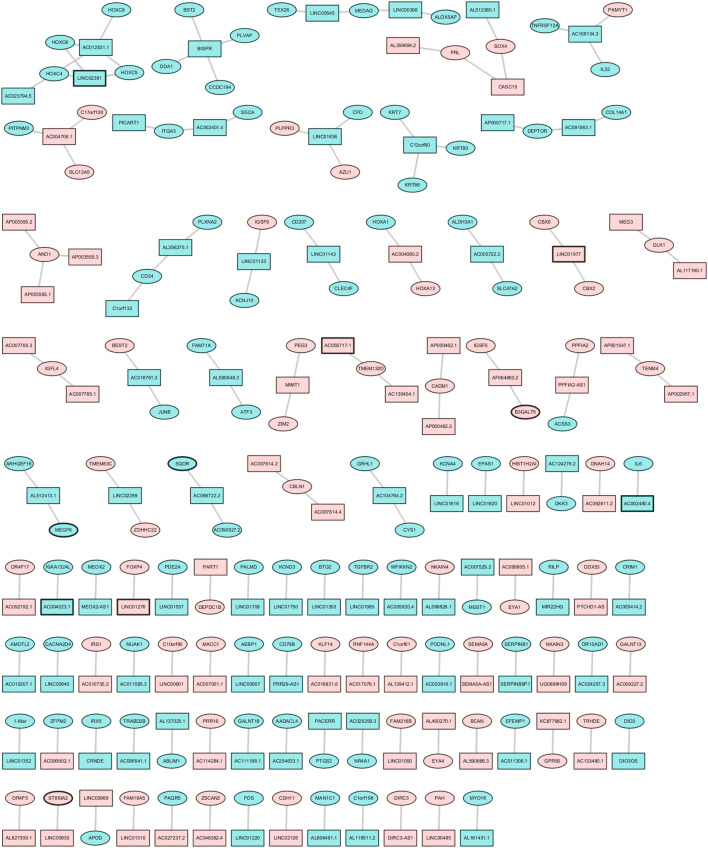
Cis-nearby-targeted DEmRNAs of DElncRNAs. There were 409 DElncRNA–nearby-targeted DEmRNA pairs, including 118 DElncRNAs and 136 DEmRNAs.

**FIGURE 5:**
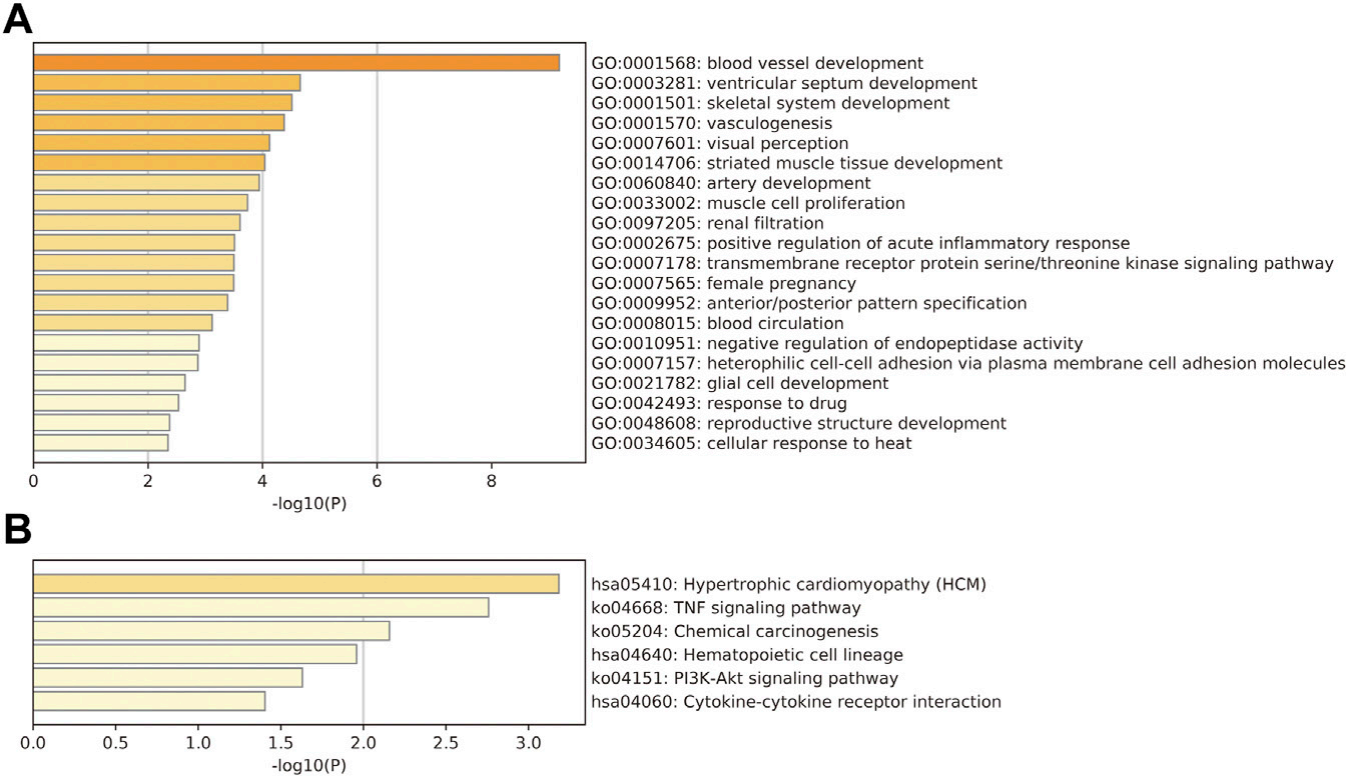
Functional annotation analysis for the validated cis-nearby-targeted DEmRNAs of DElncRNA gathering genes. **(A)** GO analysis. **(B)** KEGG pathway analysis. The vertical axis shows the annotated functions of the target genes. The horizontal axes show the enrichment score (−log10 transformed *p* value) and the gene number of each cluster, respectively. Only the top 10 significantly enriched clusters are included.

There were 409 DElncRNA–nearby-targeted DEmRNA pairs, including 118 DElncRNAs and 136 DEmRNAs (Figure 4). The top two DElncRNAs with the most nearby DEmRNAs were BISPR and AC012531.1, which contained four DElncRNAs and four nearby DEmRNAs, respectively. Blood vessel development (*p* = 6.61E-10), ventricular septum development (*p* = 2.21E-5), and skeletal system development (*p* = 3.08E-06) were significantly enriched GO terms (Figure 5A). Hypertrophic cardiomyopathy (*p* = 6.54E-04), the TNF signaling pathway (*p* = 1.75E-03), and chemical carcinogenesis (*p* = 6.95E-03) were significantly enriched KEGG pathways (Figure 5B).

## Discussion

UL, as a common disease in women, occurs in women of reproductive age (Segars and Al-Hendy, 2017; Mathew et al., 2021). Most of the patients with UL do not affect the physical health of women, but there is a need for surgery for UL patients with fertility requirements, which cause female menorrhagia, pelvic pain, and compression symptoms. However, the pathogenesis of UL is not fully understood. Therefore, it is necessary to identify the molecular mechanisms of disease progression that are useful in the diagnosis and treatment of UL (Segars and Al-Hendy, 2017; Ulin et al., 2020; Yuan et al., 2021). Recently, the abnormal expression of lncRNAs and mRNAs has been verified in various diseases, and the growing evidence indicates that lncRNAs and mRNAs are involved in the progression of diseases (Nothnick, 2016; Jain et al., 2022; Lin et al., 2022). LncRNAs are a class of non-coding RNAs with more than 200 nucleotides that are considered to be a transcriptional byproduct and do not encode proteins (Wu et al., 2021). In these years, lncRNAs, which were considered as byproducts of RNA polymerase II, have emerged to play important roles in regulating various physiological and pathological processes (Jain et al., 2022; Lin et al., 2022). In addition, more and more studies have demonstrated that lncRNAs are closely associated with the occurrence and development of tumors, aging, and inflammation, with the rapid development of molecular biology and genetic diagnostic techniques (Klapproth et al., 2021; Zhao et al., 2021). A great number of studies have found the dysregulation of lncRNAs and mRNAs involved in many cell-signaling pathways and participated in the occurrence and development of UL (Nothnick, 2016; Lin et al., 2022). For example, the increased lncRNA XIST in UL could lead to reduced expression of miR-29c and miR-200c by targeting COL1A1, COL3A1, and FN1 (Chuang et al., 2021). In addition, lncRNA AL445665.1–4 was found to be involved in the development of UL through interacting with miR-146b-5p (Yang et al., 2019). At last, lncRNA APTR promoted the proliferation of UL cells through the Wnt pathway by targeting ERα (Zhou et al., 2021). All this indicated that lncRNAs and mRNAs could be used as novel biomarkers for the diagnosis and treatment of UL.

Generally speaking, lncRNAs activate or inhibit gene expression through the recruitment of various remodeling complexes to a gene promoter, ultimately affecting epigenetics (Klapproth et al., 2021; Lin et al., 2022). Accumulating evidence showed that different biological functions of lncRNAs mainly depend on their clearly different subcellular localization (Nothnick, 2016). Cytoplasmic lncRNAs could function as decoys for miRNAs, leading to the regulation of mRNA stability or translation and finally influencing signaling pathways (Chuang et al., 2021; Zhao et al., 2021). In this study, a total of 553 DElncRNAs (283 upregulated DElncRNAs and 270 downregulated DElncRNAs) and 3,293 DEmRNAs (1,632 upregulated and 1,661 downregulated DEmRNAs) were found in UL tissue samples. To further dissect the function of those target genes contained in the DElncRNA and DEmRNA networks, GO analysis revealed that mRNAs were deregulated in UL and were mainly involved in a number of signaling pathways, including blood vessel development, regulation of ion transport, and external encapsulating structure organization. KEGG pathway analysis revealed that the deregulated mRNAs were primarily enriched in signaling pathways associated with cytokine–cytokine receptor interaction, neuroactive ligand–receptor interaction, and complement and coagulation cascades. The main blood supply for uterine fibroids is from the uterine artery and its branches. Blood vessels deliver oxygen and nutrients to every part of the body. The dysfunction of blood vessel formation was responsible for the initiation and progression of diseases including cancer (Naito et al., 2020). Elevated vascular endothelial-derived growth factor (VEGF) was found in UL compared with the uterine myometrial layer, suggesting that blood vessel development may participate in the development and growth of UL (Wei et al., 2006; Tal and Segars, 2014; Naito et al., 2020).

In this study, we speculated that some lncRNAs might regulate genes in a cis-regulatory fashion. For example, LncRNA BST2 interferon-stimulated positive regulator (BISPR) may regulate the production of inflammatory mediators and promote tumorigenesis (Pham et al., 2017). Many studies reported that the BISPR was involved in multiple malignant behaviors for tumor development, including renal cell carcinoma and oral cavity cancer (Fang et al., 2014; Pham et al., 2017). Furthermore, BISPR increased the propagation of cancer cells in thyroid papillary carcinoma by inhibiting miR-21-5p (Zhang et al., 2018). In this study, we found that the top two DElncRNAs with the most nearby DEmRNAs were BISPR and AC012531.1, which contained four DElncRNAs and four nearby DEmRNAs, respectively. Blood vessel development, ventricular septum development, and skeletal system development were significantly enriched GO terms. Hypertrophic cardiomyopathy, the TNF signaling pathway, and chemical carcinogenesis were significantly enriched KEGG pathways. Taken together, our findings revealed that lncRNAs might act as ceRNAs to impact blood vessel development and hypertrophic cardiomyopathy during the development of UL.

However, our study has several limitations. First, RNA-seq and mRNA-seq were performed to predict the potential roles of lncRNAs and mRNAs by bioinformatics analysis. Although some experimentally validated gene regulatory networks appeared in our analysis, it is still necessary to validate these results with related experiments. In addition, it remains to be further validated because of the relatively small sample size, contributing to potentially limiting the statistical power to investigate the real association. Hence, a larger sample size is needed to verify our results. In this study, high-throughput RNA sequencing technology was applied to screen lncRNAs specifically expressed in UL tissues, and bioinformatics methods were used to analyze the key pathways enriched by specific lncRNAs in GO terms and KEGG pathways. In the next step, we will still expand the sample size of clinical studies, further verify the differential expression of lncRNAs and their target genes between UL tissues and surrounding normal myometrial tissues, and verify the regulation of lncRNAs on their target genes by *in vitro* cell experiments in order to discuss the pathogenesis of UL from the perspective of epigenetics and provide a preliminary theory for the prevention and treatment of UL.

## Conclusion

In summary, these research results suggest that 3,293 DEmRNAs and 553 DElncRNAs are differentially expressed in UL tissues and surrounding normal myometrial tissues. The DElncRNA–DEmRNA co-expression networks and PPI networks provide new insight into the biological processes and underlying mechanisms of UL. This study might also highlight lncRNAs as candidate-identified therapeutic and prognostic targets for UL and offer several possible mechanisms and pathogenesis of UL in the future.

## Data Availability

The data presented in the study are deposited in the SRA repository, accession number PRJNA12360444.
